# Efficacy and safety of opioid-receptor antagonists for opioid-induced constipation: a systematic review and meta-analysis

**DOI:** 10.3389/fphar.2025.1749875

**Published:** 2026-01-12

**Authors:** Yuanlin Li, Yuyuan Tu, Zihao Zhou, Defu Liao, Ziyan He, Yan Li, Boyu Li, Zhiren Liu, Zugang Zhou, Shuangchun Ai

**Affiliations:** 1 School of Health and Rehabilitation, Chengdu University of Traditional Chinese Medicine, Chengdu, China; 2 Department of Rehabilitation, Mianyang Hospital of Traditional Chinese Medicine, Mianyang, China

**Keywords:** adverse events, opioid-induced constipation, opioid-receptor antagonists, randomized controlled trials, spontaneous bowel movement

## Abstract

**Background:**

Opioid-induced constipation (OIC) is a common and serious side effect of long-term opioid analgesic therapy. As traditional laxatives often show limited efficacy, it is crucial to explore treatment strategies that effectively relieve constipation without compromising analgesic effects. In response to this clinical need, Opioid-receptor antagonists have been approved for OIC. Although new evidence has emerged in recent years, a comprehensive analysis of efficacy outcomes (such as constipation symptoms, quality of life, and satisfaction) is still lacking.

**Objective:**

To summarise and analyze evidence on the efficacy and safety of opioid-receptor antagonists in treating patients with OIC.

**Method:**

A systematic search of randomized controlled trials (RCTs) was conducted in PubMed, Embase, Web of Science, and the Cochrane Library up to 11 September 2025. A meta-analysis was carried out using RevMan and Stata, and the GRADE method was employed to evaluate the quality of evidence.

**Results:**

A total of 20 studies (22 RCTs) involving 7,761 patients were included. Opioid-receptor antagonists significantly increased the change in spontaneous bowel movement (WMD = 1.10, 95% CI: 0.74–1.46); improved the proportion of responders (RR = 1.48, 95% CI: 1.28–1.70); enhanced quality of life (WMD = −0.20, 95% CI: −0.28 to −0.12) and treatment satisfaction (WMD = −0.32, 95% CI: −0.54 to −0.10). The patient assessment of constipation symptoms questionnaire showed a minor tendency of improvement (WMD = −0.16, 95% CI: −0.31 to 0.00). The incidence of serious adverse events indicates that no statistically significant difference was observed between treatment and placebo (RR = 0.88, 95% CI: 0.74–1.05). The incidence of other adverse events was higher in the treatment group than in the placebo group (RR = 1.22, 95% CI: 1.08–1.38).

**Conclusion:**

Opioid-receptor antagonists are effective in treating patients with OIC. The risk of serious adverse events did not change statistically. The incidence of adverse events appears to increase with longer treatment duration, although this observation seems to require further validation.

**Systematic Review Registration:**

CRD420251154280.

## 1 Introduction

Opioids are widely used analgesics, particularly in chronic pain management (Akhgarandouz et al., 2024; De Giorgio et al., 2021). Despite their powerful analgesic properties, the drug side effects (such as sedation, itching, and a significant risk of addiction) can often be concerning (Farmer et al., 2019). One of the common adverse events is opioid-induced bowel dysfunction (De Giorgio et al., 2021). Opioid-induced bowel dysfunction is a manifestation of the drug’s overall impact on the gastrointestinal system (including nausea, vomiting, bloating, symptoms related to gastroesophageal reflux, and constipation), with opioid-induced constipation (OIC) being a clinical manifestation (Farmer et al., 2019).

Enteric neurons in the gastrointestinal tract control the contraction and relaxation of the intestinal muscle layer by producing various neuroactive molecules (acetylcholine, substance P, and vasoactive intestinal polypeptide, etc.) (Holzer, 2009). At the same time, certain neuroactive molecules can bind to corresponding receptors (especially µ-opioid-receptors), thereby affecting the secretion and absorption of water in the intestine (Galligan and Akbarali, 2014). Opioids can inhibit the release of neuroactive molecules from neurons, causing decreased gastrointestinal motility, increased colonic fluid absorption, and reduced secretion, leading to harder stools (Serra et al., 2024). OIC refers to the emergence of new symptoms or the worsening of pre-existing constipation symptoms after the initiation, change, or increase of opioid therapy (Drossman and Hasler, 2016). OIC affects up to 80% of patients undergoing long-term opioid therapy, a condition that severely reduces patients’ quality of life and further drives up healthcare costs (Crockett et al., 2019a; Nishie et al., 2019).

Traditional laxatives (such as polyethylene glycol, senna, bisacodyl, and others) are easily accessible and reasonably priced, making them the first-line option for the clinical management of OIC (Rao et al., 2019). The mechanism of OIC involves a constellation of abnormalities (including impaired gut motility, delayed transit, and dysregulation of fluid secretion and absorption), which limits the effectiveness of traditional laxatives for many OIC patients (Farmer et al., 2019). Additionally, studies have shown that laxative-related side effects (such as gas, bloating/fullness, and urgency) can be observed in up to 75% of patients (Emmanuel et al., 2017).

Opioid-receptor antagonists (ORA) can alleviate gastrointestinal side effects caused by opioids (Farmer et al., 2019). The U.S. Food and Drug Administration (FDA) has approved methylnaltrexone, naloxone, naloxegol, and naldemedine for treating OIC (Nee et al., 2018). Naloxone is a non-selective opioid receptor antagonist with affinity for the μ-receptor, δ, and κ-receptors (Banghart et al., 2013). Naloxone is typically used as an intravenous reversal agent for opioid overdose, and its unmodified release may lead to withdrawal symptoms (Farmer et al., 2019; Sykes, 1996). However, the oral bioavailability of naloxone is low (2%), and its half-life is relatively short (1–1.5 h) (Sanders et al., 2015). When administered orally at a specific dose in oxycodone/naloxone combinations, naloxone acts primarily locally in the gut (Leppert, 2010). As a result, the likelihood of central analgesic reversal is extremely low (Gibson and Pass, 2014). In contrast, peripherally acting μ-opioid receptor antagonists (PAMORAs) (naloxegol, methylnaltrexone, and naldemedine) selectively target peripheral μ-opioid receptors, exhibit higher oral bioavailability (Costanzini et al., 2021). PAMORAs can block μ-opioid receptors in the gastrointestinal tract without entering the central nervous system, which helps preserve the analgesic effects of opioids and avoids central withdrawal reactions (Farmer et al., 2019).

Nevertheless, new randomized controlled trials (RCTs) have been conducted on some of these drugs. We update previous research and further analyze the efficacy and safety of ORAs in the treatment of OIC. The findings of this study provide high-quality evidence that could inform the clinical use of ORAs and support the pharmacological management of patients with OIC.

## 2 Methods

This meta-analysis was registered in the PROSPERO database (ID CRD420251154280) and followed the Preferred Reporting Items for Systematic Reviews and Meta-Analyses (PRISMA) (Supplementary Table S1) (Page et al., 2021).

### 2.1 Search strategy

The search was conducted up to 11 September 2025. We searched the PubMed, Embase, Web of Science, and Cochrane databases for RCTs from the inception of their databases. The search terms included opioid-induced constipation, opioid-receptor antagonists, naldemedine, methylnaltrexone, naloxone, naloxegol, bevenopran, alvimopan, and randomized controlled trial. The search strings are detailed in Supplementary Table S2. Additionally, we manually searched the reference lists of the included literature to identify potentially relevant studies.

### 2.2 Inclusion and exclusion criteria

The selection criteria for this study were based on the PICOS framework. 1) Population: adults (age ≥18 years) who received opioid or opiate drug treatment and were diagnosed with OIC or opioid-induced bowel dysfunction with constipation; 2) Intervention: the treatment group received opioid-receptor antagonists therapy; 3) Comparison: the control group received placebo treatment; 4) Outcome: the primary outcome measures were the change from baseline of spontaneous bowel movements (SBM), the proportion of responders, the incidence of serious adverse events (SAE), and other adverse events (OAE) during the treatment period; secondary outcome measures included changes in Patient Assessment of Constipation Quality of Life (PAC-QOL), changes in the patient assessment of constipation symptoms (PAC-SYM), and changes in the PAC-QOL satisfaction domain score; studies reported at least one related outcome; 5) Study design: the study design was RCT, and both patients and researchers were blinded.

Studies that met the following criteria were excluded: 1) Conference reports, abstracts, animal studies, and duplicate studies; 2) Non-randomized controlled trials; 3) Missing or unobtainable objective data, and inability to obtain the full text after contacting the corresponding author; 4) Studies involving healthy volunteers as subjects.

### 2.3 Study selection and data extraction

EndNote software was used to manage and review the literature. Two researchers (Y L and Y T) independently screened titles/abstracts, reviewed full texts, and selected studies for inclusion. Discrepancies were resolved by a third researcher (ZZ).

We considered SBM (defined as a bowel movement without a rescue laxative taken within the past 24 h) (U.S. National Library of Medicine, 2009a) to be the same concept as rescue-free bowel movements (defined as a bowel movement where no laxatives were used during the prior 24 h) (U.S. National Library of Medicine, 2011b). For the PAC-QOL, a lower score was interpreted as a better quality of life. For the PAC-SYM, a decrease in score corresponded to an improvement in constipation symptoms. Responders were defined as those who had at least 3 weeks of SBM at least 3 times per week with at least one additional 1 SBM per week compared to baseline (Webster et al., 2017). If there were multiple time point measurements, we chose the one with the most extended duration to eliminate arbitrariness or duplicate calculations (Nishie et al., 2019).

All available data from the included studies were extracted into Microsoft Excel. The main extracted content was as follows: general information, including study title, first author’s name, year of publication, and gender ratio of the study population; intervention details, including drug name, dosage, frequency of administration, and treatment duration; outcome-related indicators and data. For literature that reported data only in graphical form, we extracted data from the images using GetData Graph Digitizer. When the standard deviation could not be obtained, we performed data conversion according to the values of the baseline and endpoint. If a study included multiple treatment groups compared to a single placebo group, we combined the data of the treatment groups.

### 2.4 Assessment of bias risk and certainty of the evidence

Two researchers (YL and YT) independently assessed the risk of bias for each study using the Cochrane tool, with discrepancies resolved by a third researcher (ZZ). The tool’s evaluation criteria include random sequence generation, allocation concealment, blinding of participants and staff, blinding of outcome assessment, incomplete outcome data, selective reporting, and other biases. Ultimately, the risk of bias for the studies was categorized as low, high, or unclear.

The GRADE approach was used to assess the certainty of evidence, which was classified as “very low,” “low,” “moderate,” or “high” (Balshem et al., 2011). RCTs were rated as high-certainty evidence. However, the quality of the studies may be downgraded due to limitations, inconsistency, indirectness, imprecision, and publication bias (Guyatt et al., 2011).

### 2.5 Statistical analysis

Subgroup analysis was conducted based on the type of medication. The statistical significance threshold was set at p < 0.05, with data integration software being RevMan 5.4 and Stata 15.0. Continuous variables were analyzed using the weighted mean difference (WMD) and 95% confidence interval (CI) as effect sizes. For binary variables, the combined risk ratio (RR) and 95% CI were used.

The chi-square test and I^2^ statistic were used to evaluate the statistical heterogeneity of the included studies. In case of P > 0.05 and I^2^ < 50%, we used a fixed-effect model. Instead, a random-effects model was used (P < 0.05 and I^2^ > 50%). A sensitivity analysis was conducted to determine the stability of the study results. In this study, we systematically assessed the robustness of the pooled effect estimate by switching statistical models and by excluding studies one by one.

### 2.6 Publication bias

When ≥10 studies were included, we used funnel plots, Egger’s test, and Begg’s test to assess potential publication bias (indicating publication bias when P < 0.05) (Begg and Mazumdar, 1994). Conversely, we used funnel plots (for continuous variables) or labbe plots (for binary variables) for assessment. If significant publication bias exists, we used the Duval and Tweedie trim-and-fill method to supplement missing studies (Irwig et al., 1998).

## 3 Results

### 3.1 Selection and inclusion of studies

**FIGURE 1:**
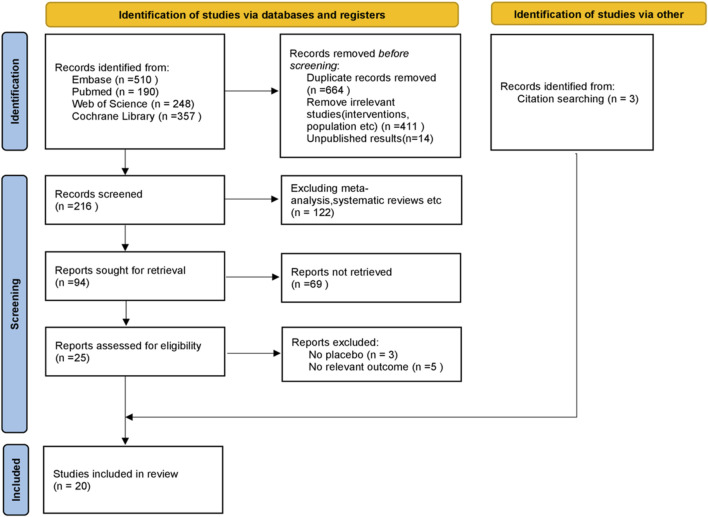
Preferred reporting items for systematic reviews and meta-analyses (PRISMA) flowchart.

A total of 1,305 studies were retrieved from four databases. Additionally, we manually retrieved three studies. After excluding duplicate studies, reviewing titles and abstracts, and reading full texts, 20 studies (22 RCTs) were included in the meta-analysis (7,761patients). Figure 1 shows the screening process and the reasons for excluding studies.

### 3.2 Research characteristics

Among 20 studies, four studies used naloxone (Akhgarandouz et al., 2024; Sanders et al., 2015; Meissner et al., 2009; U.S. National Library of Medicine, 2009a), five used naldemedine (Webster et al., 2017; Hale et al., 2017; Camilleri et al., 2021; Katakami et al., 2017a; Katakami et al., 2017b), one used alvimopan (Irving et al., 2011), four used methylnaltrexone (U.S. National Library of Medicine, 2011b; Shah et al., 2023; U.S. National Library of Medicine, 2009b; Michna et al., 2011), three used naloxegol (Webster et al., 2013; U.S. National Library of Medicine, 2011c; Chey et al., 2023), and three used bevenopran (U.S. National Library of Medicine, 2011a; U.S. National Library of Medicine, 2010; U.S. National Library of Medicine, 2012a). Two articles each reported on two trials. COMPOSE1 and COMPOSE2 were reported in one publication (Camilleri et al., 2021), COMPOSE3 and COMPOSE4 were reported in another (U.S. National Library of Medicine, 2010). The remaining 18 articles reported data from only one trial each. All studies were published in English. The detailed characteristics of each RCT are shown in Supplementary Table S3.

### 3.3 Risk of bias in included studies

**FIGURE 2:**
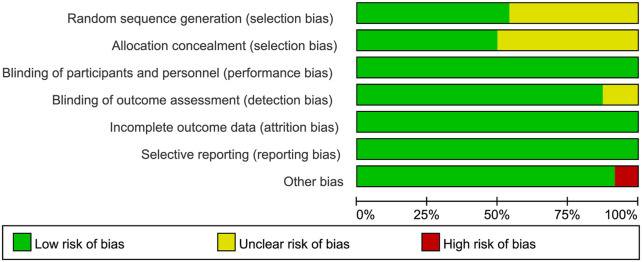
Risk of bias plot: review authors’ judgment for each risk of bias item, expressed as a percentage across all included studies.

**FIGURE 3:**
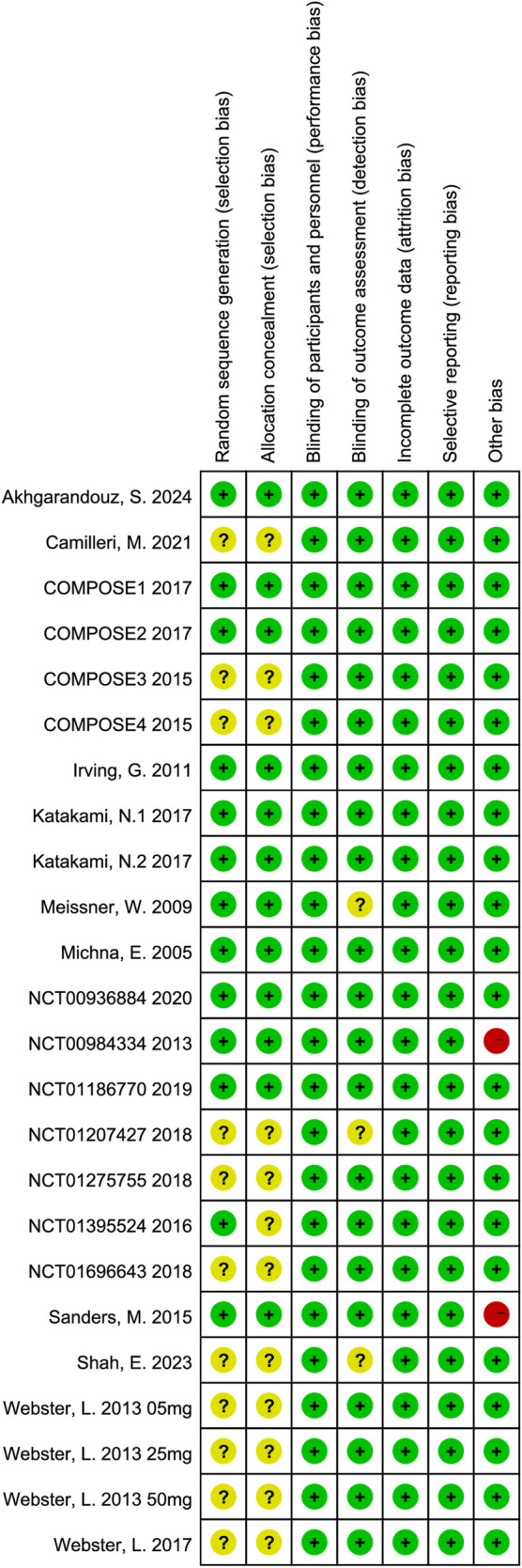
Risk of bias summary: review authors’ judgment of each risk of bias item for each included study.

The risk of bias for all included studies is shown in Figures 2, 3. All 22 included trials reported random allocation. However, 11 trials did not specify the details of random sequence generation, and 12 did not specify the details of allocation concealment, both of which were rated as unclear. Both patients and researchers in these studies were blinded to the treatment. Three trials did not clearly state whether assessors were blinded and were rated as unclear. There were no incomplete outcome data or selective reporting in any of the included studies. Among other biases, 20 RCTs were rated as low risk, and two RCTs with a small number of patients (40) were rated as high risk.

### 3.4 Analysis results

#### 3.4.1 Change from the baseline of SBM

Nine trials (1,765 patients) were included to analyze changes in weekly SBM compared to baseline (Sanders et al., 2015; U.S. National Library of Medicine, 2011a; U.S. National Library of Medicine, 2011b; Meissner et al., 2009; U.S. National Library of Medicine, 2009a; Irving et al., 2011; Webster et al., 2013; U.S. National Library of Medicine, 2010). The study found substantial heterogeneity overall (P < 0.001, I^2^ = 70.4%), and a random-effects model was used. The results showed considerable heterogeneity within the naloxone group (within-group P = 0.011, I^2^ = 78.0%), but no heterogeneity across subgroups was observed. After carefully reading the full text of the studies, the heterogeneity may stem from differences in drug dosages. Our findings are consistent with those reported in a previous study (Nishie et al., 2019). The minimum alvimopan dose was 150 mg, while the maximum dose in other groups was 20 mg, a difference of more than 7 times. The minimum dose of naloxone was 5 mg, and the maximum dose was 50 mg, representing a 10-fold difference. We excluded two studies with high drug dosages and reanalyzed the data. Using a fixed-effects model, the results showed no overall heterogeneity (P = 0.615, I^2^ = 0%). The heterogeneity within the naloxone group was reduced (P = 0.284, I^2^ = 13.0%).

**FIGURE 4:**
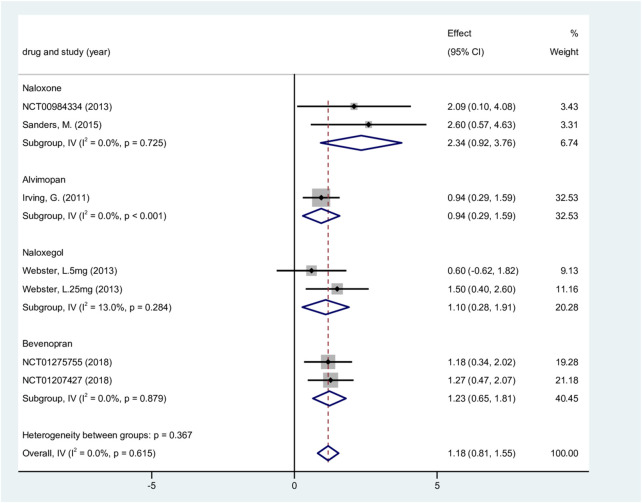
Change from the baseline of spontaneous bowel movements. CI, confidence interval.

Overall, patients receiving ORA treatment had significantly higher SBM than the placebo group (WMD, 1.18; 95% CI, 0.81–1.55; P < 0.001; Figure 4). In the subgroup analysis, significant improvements were observed for naloxone, alvimopan, naloxegol, and bevenopran. The sensitivity analysis revealed that, after excluding any single study, the combined effect size remained statistically significant. The results were minimally affected by any single study, demonstrating good stability (Supplementary Image 1). The funnel plot indicated potential publication bias (Supplementary Image 2). Two studies had to be included to achieve symmetry in the funnel plot, indicating a minor publication bias. The corrected result was WMD = 1.10 (95% CI, 0.74–1.46; P < 0.001). The adjustment resulted in a reduced effect size compared to the initial finding. This reduction indicates a potential overestimation of the treatment effect attributable to publication bias. However, the correction did not affect the statistical significance, resulting in a slight, logical decrease in value.

#### 3.4.2 Proportion of responders

The proportion of responders was analyzed using eight trials (3,573 patients) (Webster et al., 2017; Hale et al., 2017; Katakami et al., 2017a; Katakami et al., 2017b; Irving et al., 2011; Chey et al., 2023). There was substantial heterogeneity overall (P = 0.007, I^2^ = 64.2%), and a random-effects model was used. The meta-regression with drug type as a covariate revealed that drug type (particularly alvimopan) is likely the source of heterogeneity, accounting for 100% of the variance. However, due to the limited number of studies, the statistical evidence for this conclusion is not robust (P = 0.064).

**FIGURE 5:**
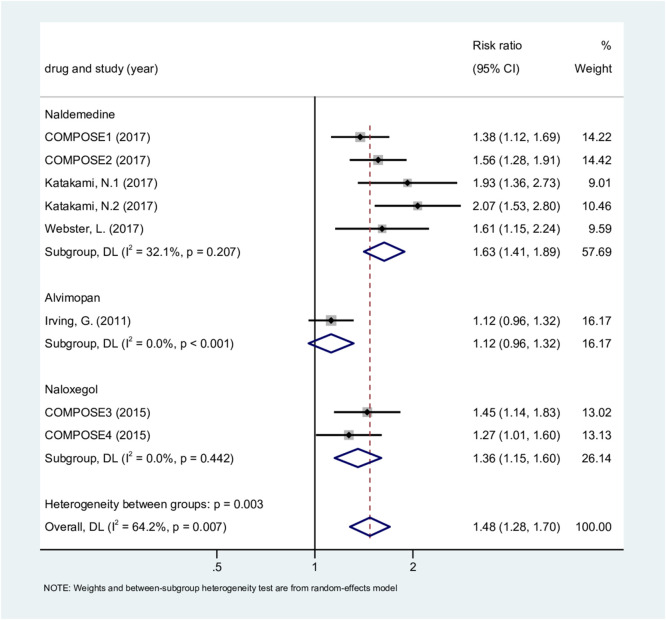
Proportion of responders.

Overall, the treatment group had a higher proportion of responders than the placebo group (RR, 1.48; 95% CI, 1.28–1.70; P < 0.001) (Figure 5). Both naldemedine and naloxegol showed significantly higher responder rates compared with placebo. For alvimopan (1 RCT; RR, 1.12; 95% CI, 0.96–1.32; P = 0.157), the point estimate suggested a possible but non-significant difference. Sensitivity analysis results indicated that the results are reliable (Supplementary Image 1). The labbe plot showed fundamental symmetry (Supplementary Image 3).

#### 3.4.3 PAC-SYM

Five trials (499 patients) were used to analyze PAC-SYM (Akhgarandouz et al., 2024; Webster et al., 2013; U.S. National Library of Medicine, 2011c). There was no overall heterogeneity (P = 0.422, I^2^ = 0%), and a fixed-effect model was used.

Patients who received ORA treatment showed a slight improvement (WMD, −0.16; 95% CI, −0.31 to −0.00; P = 0.047; Supplementary Image 4). For naloxone (1 RCT; WMD, −1.61; 95% CI, −4.06 to 0.84; P = 0.198), the change in PAC-SYM was statistically significant, but the confidence interval crossed 0. Due to its small weight in the meta-analysis (only 0.39%), its impact on the final results is negligible. When certain studies (e.g., Akhgarandouz S and NCT01395524) were excluded in the sensitivity analysis, the pooled effect estimate became non-significant (Supplementary Image 5). This observation points to limited robustness in the results. The funnel plot was generally symmetrical. (Supplementary Image 2).

#### 3.4.4 PAC-QOL

A total of six trials (1,232 patients) were included to analyze PAC-QOL (Michna et al., 2011; Webster et al., 2013; U.S. National Library of Medicine, 2011c; U.S. National Library of Medicine, 2012a). There was no heterogeneity between studies (P = 0.549, I^2^ = 0%), so a fixed-effect model was used.

Patients receiving ORA treatment had improved quality of life (WMD, −0.20; 95% CI, −0.28 to −0.12; P < 0.001; Supplementary Image 4). Sensitivity analyses indicated robustness of the results (Supplementary Image 5). The funnel plot was generally symmetrical (Supplementary Image 2).

#### 3.4.5 Satisfaction

Four trials (with 444 patients) were included to analyze changes in satisfaction levels (Webster et al., 2013; U.S. National Library of Medicine, 2011c). A fixed-effects model was used. There was low to moderate heterogeneity, which was not statistically significant (P = 0.177, I^2^ = 39.1%).

ORA treatment had a greater satisfaction improvement than placebo (WMD, −0.32; 95% CI, −0.54 to −0.10; P = 0.004; Supplementary Image 4). Sensitivity analyses indicated that the overall effect remained significant regardless of which study was excluded (Supplementary Image 5). The funnel plot was essentially symmetrical (Supplementary Image 2).

#### 3.4.6 SAE

SAE was analyzed using data from 20 studies (7,746 patients). The fixed-effect model showed consistent homogeneity among the studies (P = 0.153, I^2^ = 24.7%) (U.S. National Library of Medicine, 2011a; U.S. National Library of Medicine, 2011b; Webster et al., 2017; Meissner et al., 2009; U.S. National Library of Medicine, 2009a; Hale et al., 2017; Camilleri et al., 2021; Katakami et al., 2017a; Irving et al., 2011; U.S. National Library of Medicine, 2009b; Webster et al., 2013; U.S. National Library of Medicine, 2011c; Chey et al., 2023; U.S. National Library of Medicine, 2010; U.S. National Library of Medicine, 2012a).

**FIGURE 6:**
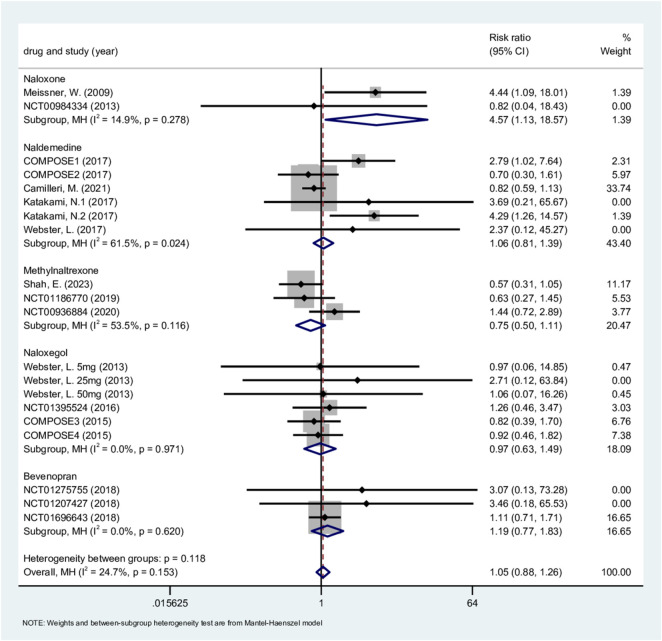
Serious adverse events.

The results showed no statistically significant difference in the incidence of adverse events between the treatment and placebo groups (RR, 1.05; 95% CI, 0.88–1.26; P = 0.593; Figure 6). In the subgroup analysis, naloxone was associated with a significantly increased incidence of SAE (2 RCTs; RR, 4.57; 95% CI, 1.13–18.57; P = 0.033). In contrast, the incidence of SAE showed no statistically significant difference from placebo for naldemedine (6 RCTs; RR, 1.06; 95% CI, 0.81–1.39; P = 0.666), bevenopran (3 RCTs; RR, 1.19; 95% CI, 0.88–1.26; P = 0.433), methylnaltrexone (3 RCTs; RR, 0.75; 95% CI, 0.50–1.11; P = 0.152), or bevenopran (6 RCTs; RR, 0.97; 95% CI, 0.63–1.49; P = 0.878). Sensitivity analyses indicate that the results are robust (Supplementary Image 1). The labbe plot indicated potential bias, with Egger’s test p = 0.027 and Begg’s test p = 0.098(Supplementary Image 3). We relied on Egger’s test results, which indicated the presence of publication bias. Seven studies were required to achieve symmetry in the labbe plot. The corrected results (RR, 0.89; 95% CI, 0.66–1.19; P = 0.416) suggested no meaningful difference in SAE risk between treatment and placebo groups. The pattern is consistent with the potential underrepresentation of smaller studies with favorable safety outcomes. We can confidently state that the core conclusion “there is no significant statistical difference in the incidence of SAE between the two groups” is powerful.

#### 3.4.7 OAE

The analysis of OAE included 17 studies (7,126 patients) (U.S. National Library of Medicine, 2011a; U.S. National Library of Medicine, 2011b; Webster et al., 2017; U.S. National Library of Medicine, 2009a; Hale et al., 2017; Camilleri et al., 2021; Shah et al., 2023; U.S. National Library of Medicine, 2009b; Webster et al., 2013; U.S. National Library of Medicine, 2011c; Chey et al., 2023; U.S. National Library of Medicine, 2010; U.S. National Library of Medicine, 2012a). Using a random-effects model, the overall results showed moderate heterogeneity (P = 0.001, I^2^ = 59.3%). The group receiving naldemedine exhibited moderate heterogeneity but did not reach statistical significance (P = 0.075, I^2^ = 56.6%). The methylnaltrexone group (P = 0.241, I^2^ = 29.8%) and the naloxegol group (P = 0.342, I^2^ = 11.5%) showed low heterogeneity. The bevenopran group showed no heterogeneity (P = 0.931, I^2^ = 0%). The moderate heterogeneity suggested that there may be other sources of variation. One study reported an increased incidence of adverse events in extension studies following 2-week trials, which appears to be related to treatment duration (Katakami et al., 2017a). Our regression analysis based on treatment duration showed that that this could explain 77.10% of the variance (P = 0.032, Adj *R* ^2^ = 77.10%, I^2^_res = 28.68%). We also performed a regression analysis by drug type to validate the rationale for subgroup analysis based on this factor. The results indicated that drug type could explain all sources of heterogeneity (P = 0.018, Adj *R* ^2^ = 100%, I^2^_res = 22.81%). Therefore, drug type is the primary factor driving between-study heterogeneity. The likely reason why treatment duration also explains a considerable proportion of heterogeneity is that clinical trials for different drugs were designed with different standard treatment cycles. For example, trials for methylnaltrexone lasted 2 or 4 weeks, whereas trials for naldemedine lasted 4, 12, and 52 weeks.

**FIGURE 7:**
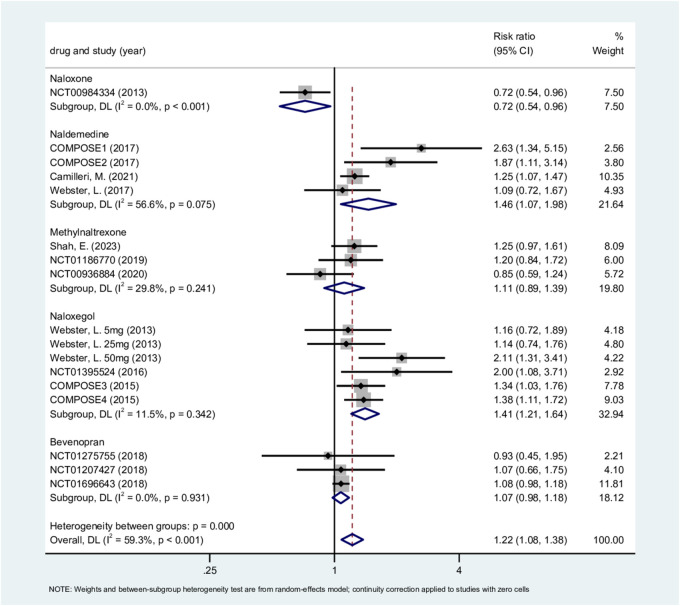
Other adverse events.

The incidence of OAE in the ORA treatment group was 1.22 times higher than in the placebo group (RR, 1.22; 95% CI, 1.08–1.38; P = 0.001; Figure 7). The naloxone group (1 RCT; RR, 0.72; 95% CI, 0.54–0.96; P = 0.023) showed a lower incidence of OAE compared to placebo. The naldemedine group (4 RCTs; RR, 1.46; 95% CI, 1.07–1.98; P = 0.016) and the naloxegol group (6 RCTs; RR, 1.41; 95% CI, 1.21–1.64; P < 0.001) both indicated a significant increase in incidence of OAE. The methylnaltrexone group (3 RCTs; RR, 1.11; 95% CI, 0.89–1.39; P = 0.338) and bevenopran (3 RCTs; RR, 1.07; 95% CI, 0.98–1.18; P = 0.131) suggested no significant increased incidence of OAE. Sensitivity analysis results showed that excluding any single study did not significantly change the estimated effect size and confidence intervals (Supplementary Image 1). The labbe plot was roughly symmetrical. Neither Egger’s test (p = 0.173) nor Begg’s test (p = 0.484) indicated publication bias (Supplementary Image 3).

### 3.5 Certainty of the evidence

Most outcome indicators (SBM, responder rates, PAC-QOL, OAE) met high GRADE standards. Satisfaction levels (as most studies did not provide detailed randomization methods) and SAEs (with high heterogeneity) were assigned a moderate level of evidence. Only PAC-SYM received a low GRADE rating (unclear randomization procedures and limited robustness of the pooled effect). The GRADE rating results are presented in Supplementary Table S4.

## 4 Discussion

This study examined six drugs (including naloxone, naldemedine, methylnaltrexone, alvimopan, naloxegol, and bevenopran). With the exception of naloxone, all other agents included in this review are PAMORAs.

### 4.1 Efficacy of the drug

In the analysis of SBM changes, we found that naloxegol showed greater improvement at higher doses in the 5–50 mg range (Webster et al., 2013). Similarly, this effect was seen with methylnaltrexone (150–450 mg) (U.S. National Library of Medicine, 2011b), naloxone (2.5–20 mg) (U.S. National Library of Medicine, 2009a; Sanders et al., 2015), and bevenopran (0.1–0.25 mg) (U.S. National Library of Medicine, 2010). However, this effect was not found in the alvimopan group (Irving et al., 2011). Kistemaker found no significant difference in short-term and medium-term responses between low doses (0.15 mg/kg) and high doses (0.30 mg/kg) of methylnaltrexone, despite the low quality of evidence (Kistemaker et al., 2024). In contrast, Rauck’s study demonstrated that 450 mg of methylnaltrexone was superior to 300 mg in terms of efficacy, and this effect was maintained throughout the treatment period (Rauck et al., 2017). Therefore, we are currently unable to establish a clear dose-efficacy correlation, and further high-quality RCTs are needed for validation.

In terms of the proportion of responders, there appears to be a difference in efficacy among the different drugs. The meta-analysis suggests that alvimopan may be inferior to other drugs in improving outcomes. Only the 0.5 mg (BID) dose showed a non-statistically significant trend towards improvement. However, more evidence is required in the future. This finding contrasts with the substantial efficacy reported in another vital study (Jansen et al., 2011). Irving explained that the unusually high placebo response rate and the differences in baseline severity of constipation among patients are the main reasons (Irving et al., 2011). Two factors may have collectively led to the “dilution” of the treatment effect of alvimopan. It makes alvimopan’s efficacy difficult to manifest (Irving et al., 2011).

During the follow-up period without treatment, the improvement in SBM decreased. In PAC-QOL, improvement relative to baseline persisted but also declined. Similarly, another study indicated that the responder rate significantly increased in OIC patients after receiving drug treatment, but then returned to near baseline levels after discontinuation (Webster et al., 2008). These suggest that patients require ongoing treatment to achieve sustained improvement (Sanders et al., 2015). Multiple studies have reported that the efficacy of ORA in treating OIC is long-lasting and well-tolerated, with patients not developing tolerance, thereby maintaining efficacy (Camilleri et al., 2021; Webster et al., 2008). It means that ORAs may be a viable option for long-term pharmacotherapy in OIC patients.

### 4.2 Safety of the drug

The most adverse events occurring during treatment were gastrointestinal reactions, which occurred at a higher rate than in the placebo group. Nevertheless, the severity was mostly mild to moderate. Gastrointestinal adverse reactions mainly manifested as diarrhea and abdominal pain (Meissner et al., 2009; Sanders et al., 2015; Hale et al., 2017; Katakami et al., 2017a; Michna et al., 2011; Chey et al., 2023; Attal et al., 1990; Paulson et al., 2005). The incidence of diarrhea increases with increasing drug dosage (Webster et al., 2017; Meissner et al., 2009; Katakami et al., 2017a). However, for most patients, diarrhea is transient and is often regarded as a “transient hypermotility”. Some researchers also believe that the gastrointestinal events experienced by patients may primarily stem from the impact on the intestines (Sanders et al., 2015; Katakami et al., 2017a). For example, a pooled analysis of Phase III study data revealed that 10.3% of patients discontinued the drug due to abdominal pain or diarrhea, compared to 5% in the placebo group (Sykes, 1996). In a pooled analysis of naldemedine, the incidence of gastrointestinal adverse events in the drug treatment group was higher than that in the placebo group (Hale et al., 2017). These findings suggest that the high incidence of abdominal pain and diarrhea may be due to the drug’s peripheral antagonistic effects on the gastrointestinal tract (Sanders et al., 2015; Katakami et al., 2017a).

Gastrointestinal perforation associated with PAMORAs is reported to be a rare occurrence (Mehta et al., 2025). ORA does not inherently increase the risk of perforation. Instead, the risk may be associated with pre-existing gastrointestinal vulnerability or concomitant medications in susceptible patients (Yokota et al., 2024). For instance, chronic opioid use can induce intestinal inflammation and alter permeability (Lacy and Cangemi, 2024). Furthermore, retained feces may lead to ischemic necrosis of the bowel wall (Farmer et al., 2019). In patients with a compromised intestinal wall, the increased motility and intraluminal pressure following PAMORA administration could potentially contribute to severe distension or perforation (Yang and Ni, 2008). Concomitant use of medications such as anti-VEGF therapies (e.g., bevacizumab) or anti-inflammatory agents may also increase the risk of gastrointestinal perforation. The annual reporting rates of fatal or life-threatening gastrointestinal perforation events for various PAMORAs in the FDA Adverse Event Reporting System were relatively similar (Mehta et al., 2025). Therefore, Italian experts recommend that close monitoring of the condition and adverse reactions is essential during ORA treatment (Varrassi et al., 2024). To avoid serious complications, three drugs (including naldemedine, methylnaltrexone, and naloxegol) are contraindicated in patients with known or suspected gastrointestinal obstruction or those at risk of recurrent obstruction (Food and Drug Administration, 2018; Food and Drug Administration, 2020; Food and Drug Administration, 2025).

Some drugs had a dose-dependent effect, with the incidence of AEs increasing with higher doses. For example, the incidence of AEs with 50 mg naloxegol was higher than with placebo, whereas the incidence with 5 or 25 mg was comparable. Naloxone and naldemedine also exhibited a dose-dependent effect.

This study showed that treatment duration may be associated with the incidence of OAE, but not with SAE (P = 0.634). For the incidence of OAE, a clear trend of increasing incidence was observed in the larger subgroup studies at 4 and 12 weeks. The incidence of OAE increased by 22% at 4 weeks (7 RCTs) and by 54% at 12 weeks (5 RCTs). In contrast, treatment for 3 weeks (1 RCT) showed a lower incidence of OAE compared to placebo. However, it should not be interpreted as conclusive evidence of protective effects for short-term treatment. Furthermore, a significant confounding factor in the current evidence was the lack of long-term study data for certain drugs. For example, the trial durations for naloxegol and methylnaltrexone were all within 4 weeks. The observed impact on the incidence of OAE might stem from either an interaction between the drug and treatment duration or from the specific properties of individual drugs, necessitating future investigation. Clinical decision-making must weigh benefits against risks. For medium-to long-term treatment (≥4 weeks), patients should be informed of the potential increase in the incidence of OAE, and drug selection should consider existing safety evidence. For patients planning treatment beyond 12 weeks, prioritizing drugs with established long-term safety data (e.g., naldemedine and naloxegol) is a more prudent approach.

It is noteworthy that some studies using sustained-release naloxone solution showed improvement in symptoms among OIC patients, but this was accompanied by a reduction in analgesia and opioid withdrawal symptoms (Attal et al., 1990; Tsuruoka and Willis, 1998). Patients on long-term opioid therapy are more likely to experience withdrawal symptoms (Tsuruoka and Willis, 1998; Berry and Dunn, 2024). Additionally, the confidence interval for the naloxone subgroup in this study was wide. Therefore, the estimate should be considered highly imprecise, as it is primarily driven by a single early study (2009) (Meissner et al., 2009). Additionally, considering the limited number of studies (only two), this signal is more likely to stem from chance or specific study limitations rather than an inherent risk associated with naloxone. Therefore, the results of the naloxone subgroup do not affect the overall conclusion: the incidence of adverse events with ORA showed no significant difference compared to placebo.

### 4.3 Selection of the drug

In the 2019 American Gastroenterological Association Institute guidelines for the management of OIC, naldemedine is recommended as first-line therapy for patients with an inadequate response to laxatives (strong recommendation, high-quality evidence) (Crockett et al., 2019b). A 2020 network analysis showed that naloxegol had the highest efficacy in terms of responder rates, while naloxone demonstrated better improvement in SBM (Ouyang et al., 2020). In another study, naloxegol produced more SBMs compared to alvimopan and naldemedine (but did not include naloxone) (Rekatsina et al., 2021). Combining the results of this analysis, naloxegol, naloxone, and naldemedine seem to be more efficacious. However, there are currently no head-to-head clinical studies for ORA, making it impossible to directly compare the efficacy of different medications (Ouyang et al., 2020; Pergolizzi et al., 2020). Ouyang R’s research demonstrated that naloxone is very unlikely to cause adverse events (Ouyang et al., 2020). In addition to considering the effectiveness and safety of the drugs, economic cost is also a concern for patients. The clinical application of naldemedine, naloxegol, and methylnaltrexone may be limited by their high economic costs (Crockett et al., 2019b). However, for patients with advanced disease, subcutaneous injection of methylnaltrexone may provide more benefits (Rekatsina et al., 2021; Pergolizzi et al., 2020). Naldemedine was associated with a higher incidence of adverse events compared to placebo in the cancer patient subgroup (Song et al., 2019). Although naldemedine is not recommended in severe hepatic impairment, naldemedine has no restrictions in patients with renal impairment (Rekatsina et al., 2021). Moreover, its safety has been demonstrated in long-term treatment studies lasting up to 52 weeks in this study. In summary, naldemedine is more suitable for non-cancer patients who require long-term oral medication (Rekatsina et al., 2021).

### 4.4 Limitations

The study has several limitations. Firstly, the funnel plot shows asymmetry between the change in SBM and the incidence of SAE, suggesting publication bias. The existence of publication bias implies a potential overestimation of the treatment effect. The adjusted results continued to support the conclusion that ORA significantly increased SBM, consistent with findings from another study (Nishie et al., 2019). Meanwhile, the incidence of SAE showed no significant difference compared to placebo, raising the possibility of an underrepresentation of smaller studies reporting better safety profiles. During the literature selection process, we discovered that some trials had been completed but had not yet uploaded their data (U.S. National Library of Me dicine, 2003; U.S. National Library of Medicine, 2012b; U.S. National Library of Medicine, 2012c; U.S. National Library of Medicine, 2017; U.S. National Library of Medicine, 2019; U.S. National Library of Medicine, 2024). Secondly, some included studies were rated as unclear regarding random allocation methods and allocation concealment. It suggests that inadequately reported randomization procedures may introduce selection bias, which can bias effect estimates upward. Thirdly, the small number of included studies on satisfaction may indicate limited strength of evidence.

## 5 Conclusion

In summary, this study systematically reviews the efficacy and safety of opioid-receptor antagonists in the treatment of OIC. The meta-analysis reveals that ORA can significantly improve weekly SBM, proportion of responders, PAC-QOL, and satisfaction in patients with OIC. Although there is an overall increase in OAE (mainly gastrointestinal), SAE rates did not differ significantly between groups. The study also identified the effects of drug type and intervention duration on the incidence of OAE. However, both the change in SBM and the incidence of SAE may be subject to publication bias. Meanwhile, the findings are limited by the insufficient reporting of randomization and allocation concealment in some of the included studies. These conclusions require further validation through more methodologically rigorous, well-reported large-scale randomized controlled trials. This study provides information that may support the clinical selection of opioid-receptor antagonists and the development of personalized management strategies for OIC patients.

## Data Availability

The original contributions presented in the study are included in the article/Supplementary Material, further inquiries can be directed to the corresponding authors.
