# Supplementary material for: Efficacy and safety of opioid-receptor antagonists for opioid-induced constipation: a systematic review and meta-analysis
Source: Front Pharmacol. 2026 Jan 12;16:1749875. doi: 10.3389/fphar.2025.1749875 (PMC12832312; doi:10.3389/fphar.2025.1749875)
Supplement: Supplementary file 6 [file Table2.docx]

pubmed

| #1 | ("Opioid-Induced Constipation"[Mesh]) | 156 |
| --- | --- | --- |
| #2 | ((((((((((((((Constipation, Opioid-Induced[Title/Abstract]) OR (Opioid Induced Constipation[Title/Abstract])) OR (Opioid-Induced Constipations[Title/Abstract])) OR (Opiate-Induced Constipation[Title/Abstract])) OR (Constipation, Opiate-Induced[Title/Abstract])) OR (Opiate Induced Constipation[Title/Abstract])) OR (Opiate-Induced Constipations[Title/Abstract])) OR (Narcotic Bowel Syndrome[Title/Abstract])) OR (Bowel Syndrome, Narcotic[Title/Abstract])) OR (Narcotic Bowel Syndromes[Title/Abstract])) OR (Opioid-Induced Bowel Dysfunction[Title/Abstract])) OR (Bowel Dysfunction, Opioid-Induced[Title/Abstract])) OR (Dysfunction, Opioid-Induced Bowel[Title/Abstract])) OR (Opioid Induced Bowel Dysfunction[Title/Abstract])) OR (Opioid-Induced Bowel Dysfunctions[Title/Abstract]) | 951 |
| #3  #1 OR #2 | (("Opioid-Induced Constipation"[Mesh]) OR (((((((((((((((Constipation, Opioid-Induced[Title/Abstract]) OR (Opioid Induced Constipation[Title/Abstract])) OR (Opioid-Induced Constipations[Title/Abstract])) OR (Opiate-Induced Constipation[Title/Abstract])) OR (Constipation, Opiate-Induced[Title/Abstract])) OR (Opiate Induced Constipation[Title/Abstract])) OR (Opiate-Induced Constipations[Title/Abstract])) OR (Narcotic Bowel Syndrome[Title/Abstract])) OR (Bowel Syndrome, Narcotic[Title/Abstract])) OR (Narcotic Bowel Syndromes[Title/Abstract])) OR (Opioid-Induced Bowel Dysfunction[Title/Abstract])) OR (Bowel Dysfunction, Opioid-Induced[Title/Abstract])) OR (Dysfunction, Opioid-Induced Bowel[Title/Abstract])) OR (Opioid Induced Bowel Dysfunction[Title/Abstract])) OR (Opioid-Induced Bowel Dysfunctions[Title/Abstract])) | 960 |
| #4 | ("Narcotic Antagonists"[Mesh]) | 16252 |
| #5 | (((((((((((((((((((((((((((((((Antagonists, Narcotic[Title/Abstract]) OR (Opioid Receptor Antagonists[Title/Abstract])) OR (Antagonists, Opioid Receptor[Title/Abstract])) OR (Receptor Antagonists, Opioid[Title/Abstract])) OR (Opioid Receptor Antagonist[Title/Abstract])) OR (Antagonist, Opioid Receptor[Title/Abstract])) OR (Receptor Antagonist, Opioid[Title/Abstract])) OR (Narcotic Antagonist[Title/Abstract])) OR (Antagonist, Narcotic[Title/Abstract])) OR (Opioid Antagonist[Title/Abstract])) OR (Antagonist, Opioid[Title/Abstract])) OR (Opioid Antagonists[Title/Abstract])) OR (Antagonists, Opioid[Title/Abstract])) OR (Competitive Opioid Antagonists[Title/Abstract])) OR (Antagonists, Competitive Opioid[Title/Abstract])) OR (Opioid Antagonists, Competitive[Title/Abstract])) OR (Competitive Opioid Antagonist[Title/Abstract])) OR (Antagonist, Competitive Opioid[Title/Abstract])) OR (Opioid Antagonist, Competitive[Title/Abstract])) OR (Opioid Reversal Agents[Title/Abstract])) OR (Agents, Opioid Reversal[Title/Abstract])) OR (Reversal Agents, Opioid[Title/Abstract])) OR (Opioid Reversal Agent[Title/Abstract])) OR (Agent, Opioid Reversal[Title/Abstract])) OR (Reversal Agent, Opioid[Title/Abstract])) OR (methylnaltrexone[Title/Abstract])) OR (naloxone[Title/Abstract])) OR (alvimopan[Title/Abstract])) OR (naldemedine[Title/Abstract])) OR (naloxegol[Title/Abstract])) OR (bevenopran[Title/Abstract])) OR (axelopran[Title/Abstract]) | 31926 |
| #6  #4 OR #5 | ("Narcotic Antagonists"[Mesh]) OR ((((((((((((((((((((((((((((((((Antagonists, Narcotic[Title/Abstract]) OR (Opioid Receptor Antagonists[Title/Abstract])) OR (Antagonists, Opioid Receptor[Title/Abstract])) OR (Receptor Antagonists, Opioid[Title/Abstract])) OR (Opioid Receptor Antagonist[Title/Abstract])) OR (Antagonist, Opioid Receptor[Title/Abstract])) OR (Receptor Antagonist, Opioid[Title/Abstract])) OR (Narcotic Antagonist[Title/Abstract])) OR (Antagonist, Narcotic[Title/Abstract])) OR (Opioid Antagonist[Title/Abstract])) OR (Antagonist, Opioid[Title/Abstract])) OR (Opioid Antagonists[Title/Abstract])) OR (Antagonists, Opioid[Title/Abstract])) OR (Competitive Opioid Antagonists[Title/Abstract])) OR (Antagonists, Competitive Opioid[Title/Abstract])) OR (Opioid Antagonists, Competitive[Title/Abstract])) OR (Competitive Opioid Antagonist[Title/Abstract])) OR (Antagonist, Competitive Opioid[Title/Abstract])) OR (Opioid Antagonist, Competitive[Title/Abstract])) OR (Opioid Reversal Agents[Title/Abstract])) OR (Agents, Opioid Reversal[Title/Abstract])) OR (Reversal Agents, Opioid[Title/Abstract])) OR (Opioid Reversal Agent[Title/Abstract])) OR (Agent, Opioid Reversal[Title/Abstract])) OR (Reversal Agent, Opioid[Title/Abstract])) OR (methylnaltrexone[Title/Abstract])) OR (naloxone[Title/Abstract])) OR (alvimopan[Title/Abstract])) OR (naldemedine[Title/Abstract])) OR (naloxegol[Title/Abstract])) OR (bevenopran[Title/Abstract])) OR (axelopran[Title/Abstract])) | 38899 |
| #7 | randomized controlled trial[Publication Type] OR randomized[Title/Abstract] OR placebo[Title/Abstract] | 1145987 |
| #3 AND #6 AND #7 | (((("Opioid-Induced Constipation"[Mesh])) OR (((((((((((((((Constipation, Opioid-Induced[Title/Abstract]) OR (Opioid Induced Constipation[Title/Abstract])) OR (Opioid-Induced Constipations[Title/Abstract])) OR (Opiate-Induced Constipation[Title/Abstract])) OR (Constipation, Opiate-Induced[Title/Abstract])) OR (Opiate Induced Constipation[Title/Abstract])) OR (Opiate-Induced Constipations[Title/Abstract])) OR (Narcotic Bowel Syndrome[Title/Abstract])) OR (Bowel Syndrome, Narcotic[Title/Abstract])) OR (Narcotic Bowel Syndromes[Title/Abstract])) OR (Opioid-Induced Bowel Dysfunction[Title/Abstract])) OR (Bowel Dysfunction, Opioid-Induced[Title/Abstract])) OR (Dysfunction, Opioid-Induced Bowel[Title/Abstract])) OR (Opioid Induced Bowel Dysfunction[Title/Abstract])) OR (Opioid-Induced Bowel Dysfunctions[Title/Abstract]))) AND (("Narcotic Antagonists"[Mesh]) OR ((((((((((((((((((((((((((((((((Antagonists, Narcotic[Title/Abstract]) OR (Opioid Receptor Antagonists[Title/Abstract])) OR (Antagonists, Opioid Receptor[Title/Abstract])) OR (Receptor Antagonists, Opioid[Title/Abstract])) OR (Opioid Receptor Antagonist[Title/Abstract])) OR (Antagonist, Opioid Receptor[Title/Abstract])) OR (Receptor Antagonist, Opioid[Title/Abstract])) OR (Narcotic Antagonist[Title/Abstract])) OR (Antagonist, Narcotic[Title/Abstract])) OR (Opioid Antagonist[Title/Abstract])) OR (Antagonist, Opioid[Title/Abstract])) OR (Opioid Antagonists[Title/Abstract])) OR (Antagonists, Opioid[Title/Abstract])) OR (Competitive Opioid Antagonists[Title/Abstract])) OR (Antagonists, Competitive Opioid[Title/Abstract])) OR (Opioid Antagonists, Competitive[Title/Abstract])) OR (Competitive Opioid Antagonist[Title/Abstract])) OR (Antagonist, Competitive Opioid[Title/Abstract])) OR (Opioid Antagonist, Competitive[Title/Abstract])) OR (Opioid Reversal Agents[Title/Abstract])) OR (Agents, Opioid Reversal[Title/Abstract])) OR (Reversal Agents, Opioid[Title/Abstract])) OR (Opioid Reversal Agent[Title/Abstract])) OR (Agent, Opioid Reversal[Title/Abstract])) OR (Reversal Agent, Opioid[Title/Abstract])) OR (methylnaltrexone[Title/Abstract])) OR (naloxone[Title/Abstract])) OR (alvimopan[Title/Abstract])) OR (naldemedine[Title/Abstract])) OR (naloxegol[Title/Abstract])) OR (bevenopran[Title/Abstract])) OR (axelopran[Title/Abstract])))) AND (randomized controlled trial[Publication Type] OR randomized[Title/Abstract] OR placebo[Title/Abstract]) | 190 |

Embase

| #1 | 'opioid induced constipation'/exp | 763 |
| --- | --- | --- |
| #2 | 'constipation, opioid-induced':ti,ab OR 'opioid-induced constipations':ti,ab OR 'opiate-induced constipation':ti,ab OR 'constipation, opiate-induced':ti,ab OR 'opiate-induced constipations':ti,ab OR 'narcotic bowel syndrome':ti,ab OR 'bowel syndrome, narcotic':ti,ab OR 'narcotic bowel syndromes':ti,ab OR 'opioid-induced bowel dysfunction':ti,ab OR 'bowel dysfunction, opioid-induced':ti,ab OR 'dysfunction, opioid-induced bowel':ti,ab OR 'opioid induced bowel dysfunction':ti,ab OR 'opioid-induced bowel dysfunctions':ti,ab OR 'opiate induced constipation':ti,ab OR 'opioid-induced constipation':ti,ab OR 'opioid induced constipation':ti,ab | 1729 |
| #3 | #1 OR #2 | 1845 |
| #4 | 'narcotic antagonist'/exp | 85046 |
| #5 | 'antagonists, narcotic':ti,ab OR 'opioid receptor antagonists':ti,ab OR 'antagonists, opioid receptor':ti,ab OR 'receptor antagonists, opioid':ti,ab OR 'opioid receptor antagonist':ti,ab OR 'antagonist, opioid receptor':ti,ab OR 'receptor antagonist, opioid' OR 'narcotic antagonist':ti,ab OR 'antagonist, narcotic':ti,ab OR 'opioid antagonist':ti,ab OR 'antagonist, opioid':ti,ab OR 'opioid antagonists':ti,ab OR 'antagonists, opioid':ti,ab OR 'competitive opioid antagonists':ti,ab OR 'antagonists, competitive opioid':ti,ab OR 'opioid antagonists, competitive':ti,ab OR 'competitive opioid antagonist':ti,ab OR 'antagonist, competitive opioid':ti,ab OR 'opioid antagonist, competitive':ti,ab OR 'opioid reversal agents':ti,ab OR 'agents, opioid reversal':ti,ab OR 'reversal agents, opioid':ti,ab OR 'opioid reversal agent':ti,ab OR 'agent, opioid reversal':ti,ab OR 'reversal agent, opioid':ti,ab OR 'methylnaltrexone':ti,ab OR 'naloxone':ti,ab OR 'alvimopan':ti,ab OR 'naldemedine':ti,ab OR 'naloxegol':ti,ab OR 'bevenopran':ti,ab OR 'axelopran':ti,ab OR 'narcotic analgesic agent antagonist':ti,ab OR 'narcotic analgesic antagonist':ti,ab OR 'narcotic antagonism':ti,ab OR 'narcotic antagonists':ti,ab | 40623 |
| #6 | #4 OR #5 | 97571 |
| #7 | 'randomized controlled trial':it OR 'controlled clinical trial':it OR 'randomized':ti,ab OR 'randomised':ti,ab OR 'placebo':ti,ab OR 'randomly':ti,ab OR 'trial':ti,ab OR 'groups':ti,ab | 6140653 |
|  | #3 AND #6 AND #7 | 510 |

Web of Science

| #1 | TS=(Opioid-Induced Constipation OR Constipation, Opioid-Induced OR Opioid Induced Constipation OR Opioid-Induced Constipations OR Opiate-Induced Constipation OR Constipation, Opiate-Induced OR Opiate Induced Constipation OR Opiate-Induced Constipations OR Narcotic Bowel Syndrome OR Bowel Syndrome, Narcotic OR Narcotic Bowel Syndromes OR Opioid-Induced Bowel Dysfunction OR Bowel Dysfunction, Opioid-Induced OR Dysfunction, Opioid-Induced Bowel OR Opioid Induced Bowel Dysfunction OR Opioid-Induced Bowel Dysfunctions) | 1262 |
| --- | --- | --- |
| #2 | TS=(Narcotic Antagonists OR narcotic antagonist OR Antagonists, Narcotic OR Opioid Receptor Antagonists OR Antagonists, Opioid Receptor OR Receptor Antagonists, Opioid OR Opioid Receptor Antagonist OR Antagonist, Opioid Receptor OR Receptor Antagonist, Opioid OR Antagonist, Narcotic OR Opioid Antagonist OR Antagonist, Opioid OR Opioid Antagonists OR Antagonists, Opioid OR Competitive Opioid Antagonists OR Antagonists, Competitive Opioid OR Opioid Antagonists, Competitive OR Competitive Opioid Antagonist OR Antagonist, Competitive Opioid OR Opioid Antagonist, Competitive OR Opioid Reversal Agents OR Agents, Opioid Reversal OR Reversal Agents, Opioid OR Opioid Reversal Agent OR Agent, Opioid Reversal OR Reversal Agent, Opioid OR methylnaltrexone OR naloxone OR alvimopan OR naldemedine OR naloxegol OR bevenopran OR axelopran) | 14684 |
| #3 | TS=(randomized controlled trial OR randomized OR placebo) | 821773 |
|  | #1 AND #2 AND #3 | 248 |

Cochare

| #1 | MeSH descriptor: [Opioid-Induced Constipation] explode all trees | 32 |
| --- | --- | --- |
| #2 | (Opiate-Induced Constipations):ti,ab,kw OR (Bowel Dysfunction, Opioid-Induced):ti,ab,kw OR (Opioid-Induced Bowel Dysfunction):ti,ab,kw OR (Dysfunction, Opioid-Induced Bowel):ti,ab,kw OR (Opioid Induced Bowel Dysfunction):ti,ab,kw OR (Bowel Syndrome, Narcotic):ti,ab,kw OR (Narcotic Bowel Syndrome):ti,ab,kw OR (Narcotic Bowel Syndromes):ti,ab,kw OR (Constipation, Opioid-Induced):ti,ab,kw OR (Opioid Induced Constipation):ti,ab,kw OR (Opioid-Induced Constipations):ti,ab,kw OR (Opiate-Induced Constipation):ti,ab,kw OR (Opiate Induced Constipation):ti,ab,kw OR (Opiate-Induced Constipations):ti,ab,kw OR (Constipation, Opiate-Induced):ti,ab,kw | 682 |
| #3 | #1 OR #2 | 682 |
| #4 | MeSH descriptor: [Narcotic Antagonists] explode all trees | 1698 |
| #5 | (Opioid Antagonists, Competitive):ti,ab,kw OR (Antagonists, Competitive Opioid):ti,ab,kw OR (Competitive Opioid Antagonist):ti,ab,kw OR (Opioid Antagonist, Competitive):ti,ab,kw OR (Competitive Opioid Antagonists):ti,ab,kw OR (Antagonist, Competitive Opioid):ti,ab,kw OR (Opioid Reversal Agents):ti,ab,kw OR (Agents, Opioid Reversal):ti,ab,kw OR (Agent, Opioid Reversal):ti,ab,kw OR (Reversal Agents, Opioid):ti,ab,kw OR (Reversal Agent, Opioid):ti,ab,kw OR (Opioid Reversal Agent):ti,ab,kw OR (Antagonists, Narcotic):ti,ab,kw OR (Receptor Antagonist, Opioid):ti,ab,kw OR (Opioid Receptor Antagonists):ti,ab,kw OR (methylnaltrexone):ti,ab,kw OR (naloxone):ti,ab,kw OR (alvimopan):ti,ab,kw OR (naldemedine):ti,ab,kw OR (naloxegol):ti,ab,kw OR (bevenopran):ti,ab,kw OR (axelopran):ti,ab,kw | 5235 |
| #6 | #4 OR #5 | 5235 |
| #7 | MeSH descriptor: [Randomized Controlled Trial] explode all trees | 34 |
| #8 | (randomized controlled trial):ti,ab,kw OR (randomized):ti,ab,kw OR (placebo):ti,ab,kw | 1371819 |
| #9 | #7 OR #8 | 1371819 |
| #10 | #3 AND #6 AND #9 | 361 |
