# Supplementary material for: Efficacy and safety of opioid-receptor antagonists for opioid-induced constipation: a systematic review and meta-analysis
Source: Front Pharmacol. 2026 Jan 12;16:1749875. doi: 10.3389/fphar.2025.1749875 (PMC12832312; doi:10.3389/fphar.2025.1749875)
Supplement: Supplementary file 7 [file Table3.docx]

| Study | Participants | Drug dosage | Treatment period(week) | Gender  Male (%) | OIC/OIBD | Available outcome |
| --- | --- | --- | --- | --- | --- | --- |
| Akhgarandouz, S 2024 | 56 patients  (including cancer) | Naloxone  0.5,2,4mg QD | 2 | T:79.5  P:82.3 | OIC | Change PAC-SYM |
| Meissner, W 2009 | 202 patients  (including cancer) | Naloxone  10,20,40mg QD | 4 | T:36.8  P:38.0 | OIC | SAE |
| NCT00984334 | 40 noncancer  patients | Naloxone  2.5,5,10,20mg QD | 3 | T:53.1  P:25.0 | OIC | Change SBM, SAE, OAE |
| Sanders, M, 2015 | 40 noncancer  patients | Naloxone  2.5,5,10,20mg BID | 3 | T:53.1  P:25.0 | OIC | Change SBM |
| COMPOSE1 | 545 noncancer  patients | Naldemedine  0.2mg QD | 12 | T:41.0  P38.2 | OIC | Responder rate, SAE, OAE |
| COMPOSE2 | 550 noncancer  patients | Naldemedine  0.2mg QD | 12 | T:40.2  P:38.7 | OIC | Responder rate, SAE, OAE |
| Camilleri, M. 2021 | 1240 noncancer  patients | Naldemedine  0.2mg QD | 52 | T:38.3  P:35.1 | OIC | SAE, OAE |
| Katakami, N.1 2017 | 226 patients  (including cancer) | Naldemedine  0.1,0.2,0.4mg QD | 2 | T:58.8  P:60.7 | OIC | Responder rate, SAE |
| Katakami, N.2 2017 | 193 patients  (including cancer) | Naldemedine  0.2mg QD | 2 | T:60.8  P:62.5 | OIC | Responder rate, SAE |
| Webster, L. 2017 | 238 noncancer  patients | Naldemedine  0.1,0.2,0.4mg QD | 4 | T:31.1  P:26.2 | OIC | Responder rate, SAE, OAE |
| Irving, G. 2011 | 485 noncancer  patients | Alvimopan  0.5mgQD,0.5mgBID | 12 | T:35.6  P:37.0 | OIBD | Change SBM, Responder rate |
| Shah, E. 2023 | 230 patients  (including cancer) | Methylnaltrexone  8 or 12mg/kg QOD | 2 | T:51.7  P:49.1 | OIC | SAE, OAE |
| NCT01186770 | 803 noncancer  patients | Methylnaltrexone  150,300,450mg QD | 4 | T:37.7  P:35.3 | OIC | Change SBM, SAE, OAE |
| NCT00936884 | 50 patients  (including cancer) | Methylnaltrexone  0.15,8or12mg/kg QOD | 2 | T:44.0  P:56.0 | OIC | SAE, OAE |
| Michna, E. 2005 | 133 patients  (including cancer) | Methylnaltrexone  0.15mg/kg QOD | 2 | T:43.0  P:44.0 | OIC | Change PAC-QOL |
| Webster, L. 2013 | 185 noncancer  patients | Naloxegol  5,25,50mg QD | 4 | T:38.9  P:36.8 | OIC | Change SBM, Change PAC-SYM  Change PAC-QOL, SAE, OAE  Satisfaction level |
| NCT01395524 | 296 noncancer  patients | Naloxegol  12.5,25mg QD | 12 | T:38.3  P:40.8 | OIC | Change PAC-SYM, Change PAC-QOL  Satisfaction level, SAE, OAE |
| COMPOSE3 | 641 noncancer  patients | Naloxegol  12.5,25mg QD | 12 | T:43.0  P:34.6 | OIC | Responder rate, SAE, OAE |
| COMPOSE4 | 696 noncancer  patients | Naloxegol  12.5,25mg QD | 12 | T:36.2  P:37.5 | OIC | Responder rate, SAE, OAE |
| NCT01275755 | 81 noncancer  patients | Bevenopran  0.25mg QD | 4 | T:30.0  P:31.7 | OIC | Change SBM, SAE, OAE |
| NCT01207427 | 131 noncancer  patients | Bevenopran  0.1,0.25mg QD | 4 | T:54.5  P:46.5 | OIC | Change SBM, SAE, OAE |
| NCT01696643 | 700 noncancer  patients | Bevenopran  0.25mg BID | 52 | T:37.8  P:40.4 | OIC | Change PAC-QOL, SAE, OAE |
