# Supplementary material for: Efficacy and safety of opioid-receptor antagonists for opioid-induced constipation: a systematic review and meta-analysis
Source: Front Pharmacol. 2026 Jan 12;16:1749875. doi: 10.3389/fphar.2025.1749875 (PMC12832312; doi:10.3389/fphar.2025.1749875)
Supplement: Supplementary file 8 [file Table4.docx]

**Author(s):** Yuanlin Li

**Question:** Opioid receptor antagonists compared to placebo for opioid-induced constipation

**Setting:**

**Bibliography:**

| **Certainty assessment** | | | | | | | **№ of patients** | | **Effect** | | **Certainty** | **Importance** |
| --- | --- | --- | --- | --- | --- | --- | --- | --- | --- | --- | --- | --- |
| **№ of studies** | **Study design** | **Risk of bias** | **Inconsistency** | **Indirectness** | **Imprecision** | **Other considerations** | **opioid receptor antagonists** | **placebo** | **Relative (95% CI)** | **Absolute (95% CI)** |  |  |
| **change in spontaneous bowel movement** | | | | | | | | | | | | |
| 9 | randomised trials | not serious | not serious | not serious | not serious | none | 1205 | 560 | - | MD **0.85 higher** (0.61 higher to 1.1 higher) | ⨁⨁⨁⨁ High | CRITICAL |
| **Responder Rate** | | | | | | | | | | | | |
| 8 | randomised trials | not serious | not serious | not serious | not serious | none | 1135/2204 (51.5%) | 487/1369 (35.6%) | **RR 1.43** (1.32 to 1.55) | **153 more per 1,000** (from 114 more to 196 more) | ⨁⨁⨁⨁ High | IMPORTANT |
|  |  |  |  |  |  |  |  | 34.5% |  | **148 more per 1,000** (from 110 more to 190 more) |  |  |
| **PAC-SYM** | | | | | | | | | | | | |
| 5 | randomised trials | serious | not serious | not serious | serious | none | 297 | 202 | - | MD **0.16 lower** (0.31 lower to 0 ) | ⨁⨁◯◯ Low | IMPORTANT |
| **PAC-QOL** | | | | | | | | | | | | |
| 6 | randomised trials | not serious | not serious | not serious | not serious | none | 650 | 582 | - | MD **0.2 lower** (0.28 lower to 0.12 lower) | ⨁⨁⨁⨁ High | IMPORTANT |
| **Satisfaction Level** | | | | | | | | | | | | |
| 4 | randomised trials | serious | not serious | not serious | not serious | none | 260 | 184 | - | MD **0.32 lower** (0.54 lower to 0.1 lower) | ⨁⨁⨁◯ Moderate | IMPORTANT |
| **Serious Adverse Event** | | | | | | | | | | | | |
| 20 | randomised trials | not serious | not serious | not serious | not serious | publication bias strongly suspected | 272/4552 (6.0%) | 202/3199 (6.3%) | **RR 1.04** (0.87 to 1.24) | **3 more per 1,000** (from 8 fewer to 15 more) | ⨁⨁⨁◯ Moderate | CRITICAL |
|  |  |  |  |  |  |  |  | 3.6% |  | **1 more per 1,000** (from 5 fewer to 9 more) |  |  |
| **Other Adverse Events** | | | | | | | | | | | | |
| 17 | randomised trials | not serious | not serious | not serious | not serious | none | 1467/4134 (35.5%) | 918/2997 (30.6%) | **RR 1.22** (1.15 to 1.30) | **67 more per 1,000** (from 46 more to 92 more) | ⨁⨁⨁⨁ High | CRITICAL |
|  |  |  |  |  |  |  |  | 31.6% |  | **70 more per 1,000** (from 47 more to 95 more) |  |  |

**CI:** confidence interval; **MD:** mean difference; **RR:** risk ratio
